# Comparative genomics of *Drosophila *and human core promoters

**DOI:** 10.1186/gb-2006-7-7-r53

**Published:** 2006-07-07

**Authors:** Peter C FitzGerald, David Sturgill, Andrey Shyakhtenko, Brian Oliver, Charles Vinson

**Affiliations:** 1 Genome Analysis Unit, National Cancer Institute, National Institutes of Health, Bethesda, MD 20892, USA; 2 Laboratory of Cellular and Developmental Biology National Institute of Diabetes and Digestive and Kidney, National Institutes of Health, Bethesda, MD 20892, USA; 3 Laboratory of Metabolism, National Cancer Institute, National Institutes of Health, Bethesda, MD 20892, USA

## Abstract

**Background:**

The core promoter region plays a critical role in the regulation of eukaryotic gene expression. We have determined the non-random distribution of DNA sequences relative to the transcriptional start site in *Drosophila melanogaster *promoters to identify sequences that may be biologically significant. We compare these results with those obtained for human promoters.

**Results:**

We determined the distribution of all 65,536 octamer (8-mers) DNA sequences in 10,914 *Drosophila *promoters and two sets of human promoters aligned relative to the transcriptional start site. In *Drosophila*, 298 8-mers have highly significant (*p *≤ 1 × 10^-16^) non-random distributions peaking within 100 base-pairs of the transcriptional start site. These sequences were grouped into 15 DNA motifs. Ten motifs, termed directional motifs, occur only on the positive strand while the remaining five motifs, termed non-directional motifs, occur on both strands. The only directional motifs to localize in human promoters are TATA, INR, and DPE. The directional motifs were further subdivided into those precisely positioned relative to the transcriptional start site and those that are positioned more loosely relative to the transcriptional start site. Similar numbers of non-directional motifs were identified in both species and most are different. The genes associated with all 15 DNA motifs, when they occur in the peak, are enriched in specific Gene Ontology categories and show a distinct mRNA expression pattern, suggesting that there is a core promoter code in *Drosophila*.

**Conclusion:**

*Drosophila *and human promoters use different DNA sequences to regulate gene expression, supporting the idea that evolution occurs by the modulation of gene regulation.

## Abstract 2

Comparison of DNA sequence distributions in *Drosophila *and human promoters suggests that different motifs have distinct functional roles.

## Background

The regulation of eukaryotic gene expression is a complex process involving many different control mechanisms, including chromatin structure and DNA sequences that bind specific proteins [1]. For convenience, we divide DNA sequence motifs that are bound by proteins into three distinct classes: the core promoter region where the basal transcription machinery binds; motifs within the core promoter region that bind to transcription factors; and classic enhancer or silencer motifs, that function at large distances from the transcriptional start site (TSS). Two extremes of regulated gene expression may be envisioned. In one extreme, the general transcriptional machinery is identical for all promoters, and the binding of different transcription factors to the core promoter and more distant motifs recruits and regulates RNA polymerase activity to control gene expression. In the other extreme, different motifs within the core promoter direct the assembly of transcriptional machinery with different components. The latter system is used in prokaryotic systems where different sigma factors, a component of the polymerase complex, bind different motifs in the core promoter to regulate functionally related genes [2]. This type of system also operates in sex specific tissues of *Drosophila *where the germ cells express variant isoforms of the general transcriptional complex [3,4] termed core promoter selectivity factors [5]. Furthermore, genetic studies in *Drosophila *indicate that the core promoter contains information that directs tissue-specific mRNA expression [6-9].

A variety of computational methods have been used to identify DNA binding sites for transcription factors and core promoter elements in both *Drosophila *and human [10-12]. Previous full-genome-analysis of *Drosophila *core promoters has examined abundance, but not the precise positioning of motifs near the TSS. Here, we use the technique of examining non-random distribution relative to the TSS in *Drosophila melanogaster *promoter sequences to identify DNA motifs that are biologically significant. This study adds to our understanding of *Drosophila *core promoters by identifying new motifs and showing that motifs correlate with different biological functions. Comparing these results with those obtained with human indicate that the DNA motifs that localize are different except for the strand specific core promoter elements TATA, initiator element (INR), and downstream promoter element (DPE).

## Results

Genomic DNA sequences and gene annotation data for *Drosophila *and human were downloaded from the UCSC Genome Browser site [13]. Human gene annotation data were also obtained from the DBTSS [14]. For each organism, we created a dataset corresponding to the region -1,001 to +499 base-pairs (bp) relative to the annotated TSS sequences of each RefSeq gene that had an annotated 5' untranslated region (UTR) of 10 or more bp. We created two human datasets, one using the UCSC annotations and one using the DBTSS annotations.

### Distribution of mono-nucleotides is different between *Drosophila *and human promoters

**Figure 1:**
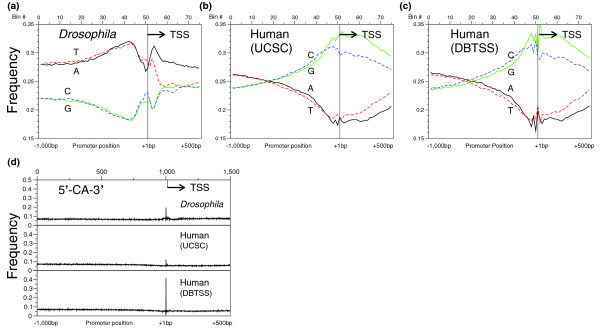
The distribution of nucleotides across *Drosophila *and human promoters. The distribution of mononucleotides across the **(a) **1,500 bp region of 10,914 *Drosophila *and **(b) **15,011 and **(c) **12,926 human promoters; the frequency of each mononucleotide is plotted against position (in 20 bp bins). The TSS occurs in bin 51 and its location is indicated. **(d) **The frequency of occurrence of the CA dinucleotide, at a single base-pair resolution across the 1,500 bp promoter region for all three datasets.

To determine the gross structure of *Drosophila *and human promoters, we determined the abundance of the four mononucleotides (1-mer; Figure 1a) across the 1,500 bp from -1,000 bp to +499 bp for 10,914 *Drosophila *promoters and compared these to distributions in 15,011 (UCSC) and 12,926 (DBTSS) human promoters (Figure 1b,c). *Drosophila *promoters are more A and T rich (56%) than human promoters (44%). In addition, *Drosophila *promoters had a peak for both A and T between -200 bp and the TSS, while the human promoters had a broad peak for both G and C centered at the TSS, suggesting a fundamental difference in global promoter architecture. The two human datasets show the same general distribution patterns, but the DBTSS set has more pronounced peaks and valleys at the TSS.

The CA dinucleotide is often associated with the TSS [15] and is often associated with a unique TSS [16]. RNA polymerase is known to prefer an adenine in the +1 position [17]. This provides an important quality control metric. A tight cluster of CA sites at the TSS would indicate that enough TSSs have been accurately assigned to permit analysis of other motifs. Figure 1d presents the CA dinucleotide distribution plotted at a single nucleotide resolution, rather than the 20 bp bin shown in Figure 1a-c. The CA distribution in both *Drosophila *and human promoters showed a spike exactly at the TSS (the A of the CA dinucleotide is at position +1 in the peak). The *Drosophila *CA spike at the TSS occurs in approximately 20% of all promoters while the spike is less pronounced in the human (UCSC) dataset (approximately 10%) and more pronounced in the human (DBTSS) dataset (approximately 40%). This CA peak is part of the initiator (INR) motif (TCAGTY) that is positioned at the TSS (see below). That CA is often present at the TSS suggests that the TSS has been appropriately assigned in many of the transcripts in both the *Drosophila *and human promoter dataset. If the CA peak is taken as a relative measure of the quality, or precise alignment, of the datasets, then the two human sets bracket the *Drosophila *set with respect to the accuracy of the positioning of the TSS.

### Distribution of all 8-mer DNA sequences in promoters

Having validated the quality of the TSS assignments, we determined the distribution of all 8-mers in the set of *Drosophila *and human putative promoters to identify potential DNA binding sites for transcription factors that are localized relative to the TSS. A clustering factor (CF), describing the presence of a peak in the distribution of each 8-mer, was calculated three ways, by examining the distribution on both strands (CF), on the positive strand (CF^+^), and on the negative strand (CF^-^). For these calculations we divided the 1,500 bp of genomic DNA, from -1,000 bp to +499 bp relative to the TSS, into 75 bins of 20 bp each (see Materials and methods).

**Figure 2:**
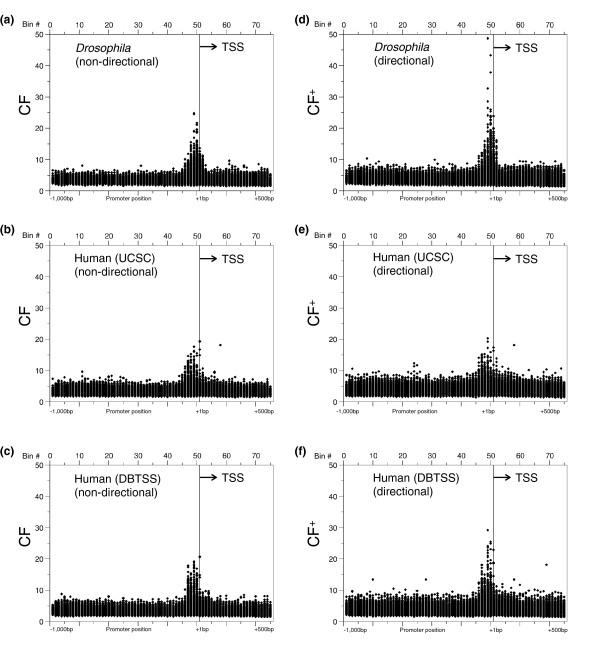
The localization of all 65,536 8-mers in *Drosophila *and human promoters. The clustering factors (CF or CF^+^) calculated for 20 bp bins plotted at the position of the most populated bin for all 65,536 8-mers. **(a) **CF for 10,914 *Drosophila *promoters; **(b) **CF for 15,011 human (UCSC) promoters; **(c) **CF for 12,926 human (DBTSS) promoters; **(d) **CF^+ ^for 10,914 *Drosophila *promoters; **(e) **CF^+ ^for 15,011 human (UCSC) promoters; **(f) **CF^+ ^for 12,926 human (DBTSS) promoters.

When CF values were plotted against the bin with the maximum number of members for the *Drosophila *and human promoters, respectively (Figure 2a-c), all distributions showed similar patterns, with a grouping of DNA sequences that peak within 100 bp of the TSS. The highest CF values for all plots is 20 to 30, indicating that these 8-mers are approximately 20 to 30 times more abundant at one position relative to the TSS than elsewhere in promoters. In contrast to the similarity in CF values, when the data were plotted for CF^+^, (Figure 2d-f), a profound difference between *Drosophila *and both human datasets was revealed. *Drosophila *8-mers have a maximum CF^+ ^value of approximately 50 while the maximum CF^+ ^for human sequences is approximately 20. This suggests that *Drosophila *has more 8-mers that occur preferentially on one strand of DNA, and that the *Drosophila *strand-dependent 8-mers have a higher degree of localization than their human counterparts. Control data, using 7th-order Markov random datasets, show a complete lack of clustering for any 8-mers for either human or *Drosophila *(data not shown).

**Figure 3:**
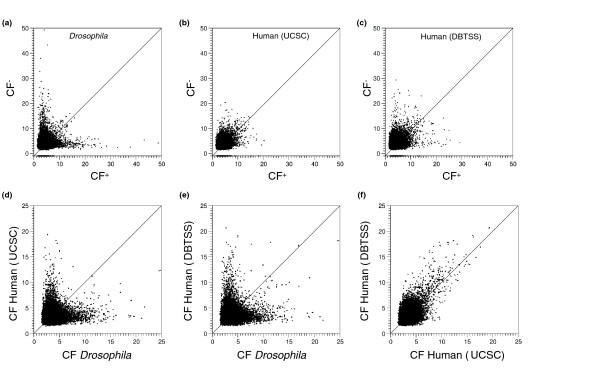
Scatter plots showing the strand dependence of 8-mer localization, and the comparison of localization between different organisms (*Drosophila *and human). The clustering factors for all 8-mers, calculated for 20 bp bins, are plotted on the positive (CF^+^) versus the negative (CF^-^) strand for **(a) ***Drosophila*, **(b) **human (UCSC), and **(c) **human (DBTSS) promoters. The 256 palindromic sequences have equivalent CF^+^/CF^- ^values but are plotted with a CF^- ^value of -1. Comparison of CF values of 8-mers for **(d) **human (UCSC) versus *Drosophila*, **(e) **human (DBTSS) versus *Drosophila*, and **(f) **human (UCSC) versus human (DBTSS). Common elements should lie along the diagonal.

To determine if an 8-mer has a peak in its distribution on only one strand of DNA, we compared the CF^+ ^with the CF on the opposite strand (CF^-^). In *Drosophila*, we identified two types of peaking 8-mers; those that peak on both strands and thus have similar CF^+ ^and CF^- ^values (termed non-directional motifs (NDMs)), and 8-mers that peak preferentially on one strand (termed directional motifs (DMs)) and thus have significantly different CF^+ ^and CF^- ^values (Figure 3a). Indeed, many motifs are randomly positioned on one strand and >20-fold enriched at a given position of the opposite strand. These two distinct types of motifs are potentially bound by proteins that have different roles in transcription regulation. The 8-mers with a high CF^+ ^but a low CF^- ^contain directional information and could be binding sites for core promoter selectivity factors. In contrast, in both human promoter sets, we observed a significant number of 8-mers that peak on both strands (Figure 3b,c), and few that preferentially peak on one strand (as shown below, these are predominantly TATA and INR-like sequences). While the human DBTSS dataset contains a greater number of DMs than does the UCSC dataset, both sets are clearly more biased toward NDM than is the *Drosophila *dataset. These data suggest that there is a significant difference in the sequence organization of promoters between these human and *Drosophila *datasets.

### *Drosophila *and human 8-mers that peak are different

Are the motifs that peak in humans similar to the motifs that peak in *Drosophila*? To answer this, we directly compared the CF values for all 8-mers between human and *Drosophila *(Figure 3d,e). The majority of 8-mers with high CF values are different between the two species. In contrast, 8-mers with the largest CF values are common between the two human datasets (Figure 3f), lending confidence to the idea that the differences between the two species are real.

### Fifteen DNA motifs that cluster in *Drosophila*

**Figure 4:**
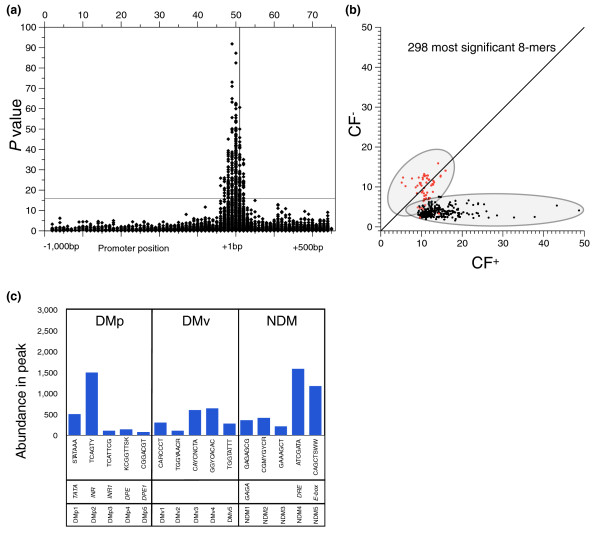
8-mer localization in *Drosophila *expressed as a probability term, and characteristics of the most statistically relevant 8-mers. **(a) **The probability term P = -log_10_(1 - *p*) for the 13,552 8-mers with a maximum bin containing ≥15 members. The 298 DNA sequences above the line at *P *= 16, a 1 in 1 × 10^16 ^(single sampling) chance of being random, were analyzed in more detail. **(b) **Clustering factors for both the positive (CF^+^) and negative strand (CF^-^) were plotted for the 298 most significant peaking 8-mers. The distribution falls into two distinct groupings; those that display a symmetric distribution on both strands (red circles) and those that cluster on only one strand (black circles). **(c) **A histogram showing the number of promoters containing each of the 15 motifs, grouped into three classes, DMp1 to 5, DMv1 to 5, and NDM1 to 5. We also present the common name and the consensus sequence.

To determine the statistical significance of the CF^+ ^values, we converted the CF^+ ^into a probability term using the 8-mer frequencies observed in the 10,914 *Drosophila *promoter dataset. The probability term, *P*, represents -log_10_(1 - *p*), where *p *is the area under the normalized curve of the distribution of CF_expt_. A high *P *value indicates that it is very unlikely that the peak for the 8-mer occurs by chance. A plot of the *P *values versus the most populated bin number (Figure 4a) shows a group of 8-mers near the TSS whose distributions are very unlikely to occur by chance. We analyzed the 298 8-mers that have a *P *value ≥ 16. All these 8-mers had peaks centered between -100 bp and +40 bp. As illustrated in Figure 4a, *P *≥ 16 is a conservative cutoff. We plotted CF^+ ^versus CF^- ^for these 298 sequences to examine their strand specific localization (Figure 4b). DMs (black circles) predominate, but NDMs (red circles) were also identified.

**Figure 5:**
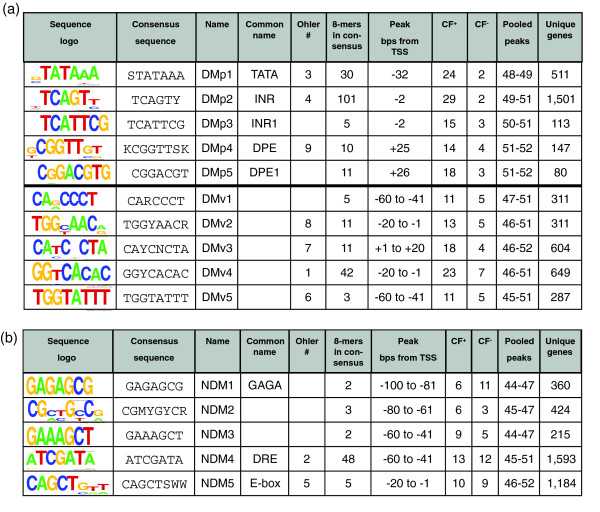
The 15 DNA motifs derived from grouping 298 octamers whose probability of having a non-random distribution was less than 1 × 10^-16^. The table is grouped into two panels. **(a) **presents the 10 directional motifs, while **(b) **shows the five non-directional motifs. We present: the sequence logo; the consensus sequence using IUPAC letters to represent degenerate bases - R (G, A), W (A, T), Y (T, C), K (G, T), M(A, C), S (G, C), N (A, T, G, C); the name assigned in this work; the common name if it exists; designations from previous work [10]; the number of 8-mers that peaked that were placed in the family; peak location as base-pairs relative to the TSS; clustering factor (CF^+^) on the positive strand; clustering factor (CF^-^) on the negative strand; the bins that were pooled to define the peak; and the unique genes in the peak.

The 298 8-mer sequences were manually grouped into 15 families and a consensus motif was determined for each family (Figure 5). The placement of an 8-mer into a particular motif was guided by: the similarity amongst DNA sequences; the shape of the distribution histogram; the peak position relative to the TSS; and whether the 8-mer was directional or non-directional. The total number of 8-mers in each of the 15 motifs varied dramatically, with over one-third of the 298 8-mers representing variations of the INR motif (TCAGTY) and 8 motifs were represented by 5 or fewer 8-mers. We determined the abundance of the 15 motifs by counting unique promoters that contained a motif in the peak (Figure 4c). A total of 6,067 promoters contain one or more of the 15 motifs. The most abundant motif is the non-directional DRE, found in 15% (1,593) of *Drosophila *promoters, followed by directional INR, found in 14% (1,501) of promoters. The least abundant motif identified, DMp5, is found in 0.7% (80) of all promoters.

**Figure 6:**
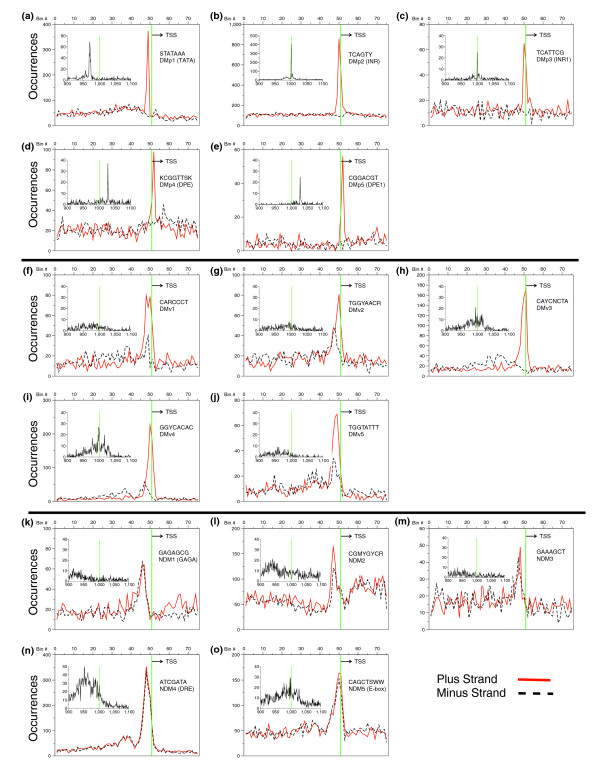
The distribution of the 15 identified motifs in *Drosophila *promoters. **(a-o) **The number of occurrences of each motif, in each 20 bp bin, for the positive strand (solid red) and the negative strand (dashed black). The inserts show the same data plotted at a single nucleotide resolution from -100 bp to +100 bp relative to the TSS. Inserts for the directional motifs (DMp1 to 5 and DMv1 to 5) show the distribution on the positive strand only, while those for the non-directional motifs (NDM1 to 5) show the distribution for both strands. (a-e) The directional motifs that have a precise localization (DMp); (f-j) the directional motifs with a variable localization (DMv); (k-o) the non-directional motifs that all have a variable localization (NDM).

Figure 6 presents the distribution of each of the 15 consensus motifs, showing the number of occurrences on each DNA strand. To gain more insight into how constrained motif position is relative to the TSS, we examined the distribution of the 15 DNA motifs at a single base-pair resolution. The inserts in Figure 6 show the single base-pair distribution plots for the motifs in the region -100 to +100 relative to the TSS. Five of the DMs (Figure 6a-e) are positioned at a single base-pair resolution relative to the TSS while the other five DMs (Figure 6f-j) and the five NDMs (Figure 6k-o) are spread across a broad region of up to 50 bp, though they all clustered near the TSS. We thus classified the DMs as either precise or variably positioned. The DMs are named DMp1 to 5 (for directional motif precise) and DMv1 to 5 (for directional motif variable). The NDMs are named NDM1 to 5. Where a motif has a previous common name we use that name, for example, DMp1 is TATA, DMp2 is INR, DMp4 and DMp5 are DPE-like, NDM1 is GAGA and NDM4 is downstream responsive element (DRE). The single base-pair resolution plots not only reveal the precise versus variable positioning of the motifs, they also reveal the power of the initial analysis based on 20 bp bins. Many of the motifs (DMvs and NDMs) would not have been identified at a single base-pair resolution. Also, the number of promoters identified that contain a specific motif is much greater at a 20 bp resolution than a 1 bp resolution (for example, for INR there are approximately 1,500 versus approximately 400).

**Figure 7:**
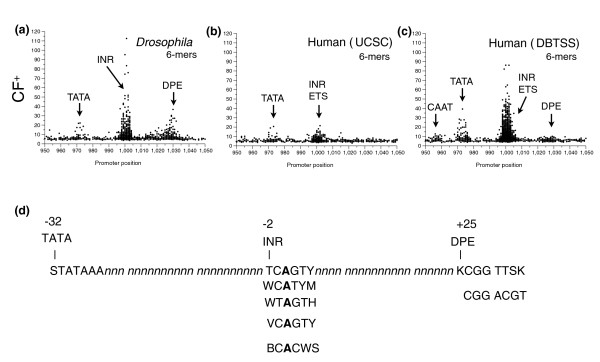
The localization, on the positive strand, of all 4,096 6-mers in *Drosophila *and human promoters. Clustering factor (CF^+^) for the positive strand, plotted at a single base-pair resolution, at the position of the most populated bp, for all 4,096 6-mers. **(a) **CF^+ ^from 10,914 *Drosophila *promoters; **(b) **CF^+ ^from 15,011 human (UCSC); **(c) **CF^+ ^from 12,926 human (DBTSS) promoters; **(d) **the exact placement of *Drosophila *TATA, INR variants, and DPE variants relative to each other. The sequence is broken into 10 bp segments.

To further examine the localization of DNA sequences at a single base-pair resolution, we examined the CF^+ ^values of all 6-mers for both *Drosophila *and human promoters (Figure 7). We chose 6-mers to produce enough occurrences at each base pair position to be able to determine peaks reliably. The *Drosophila *data (Figure 7a) showed three distinct regions in which individual 6-mers were preferentially localized. Examination of the DNA sequences that cluster around each of these three positions indicated they can be grouped into a single motif that is localized at a specific base-pair position relative to the TSS. The three motifs are TATA, INR and DPE. Where promoters have two of these motifs, they are precisely positioned relative to each other (Figure 7d).

The clustering of 6-mers at a single base-pair resolution in the UCSC human promoters showed generally lower CF^+ ^values and only two peaks corresponding to the TATA and INR positions (Figure 7b). While the DBTSS dataset (Figure 7c) showed more pronounced peaks than the UCSC dataset, it still failed to show a clear DPE peak. Examination of the sequences localized under the main human (DBTSS) peaks produced a result similar to that seen form *Drosophila*. The sequences lying under the TATA peak were exclusively TATA-like sequences. The sequences under the INR peak represented INR variants localized exactly at the TSS and other NDMs, predominantly erythroblast transformation specific (ETS), localized close to the TSS. However, the variety of INR sequences that localized in the human dataset was greater than that seen for the *Drosophila *data. Attempts to identify distinct human INR motifs six nucleotides or greater were unsuccessful due to the wide degeneracy in sequences that surround the prominent central CA core.

### Comparison of *Drosophila *and human motifs that peak

**Figure 8:**
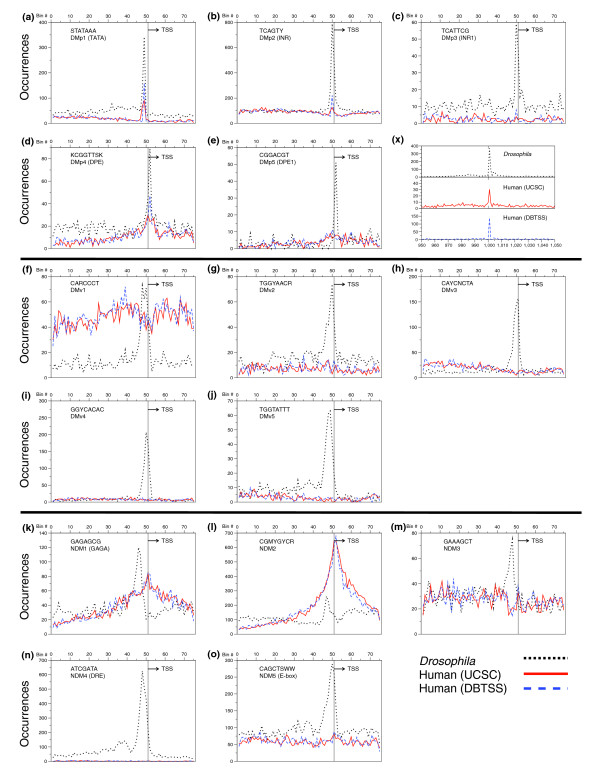
The distribution of 15 '*Drosophila *specific' motifs in *Drosophila *and human promoters. **(a-o) **The number of occurrences of each of the 15 identified *Drosophila *motifs in each 20 bp bin for *Drosophila *(dotted black), human (UCSC; solid red) and human (DBTSS; dashed blue) promoters. For the ten directional motifs, only the occurrences on the positive strand are represented. For the five non-directional elements, the occurrences on both the positive and negative strand are represented. **(x) **The distributions of the INR motif (TGACTY), from -100 to +100, for both *Drosophila *and human promoters at a single base-pair resolution. The number of occurrences of each element has been normalized, based on a dataset of 10,000 promoters, to compensate for the different sizes of the datasets.

**Figure 9:**
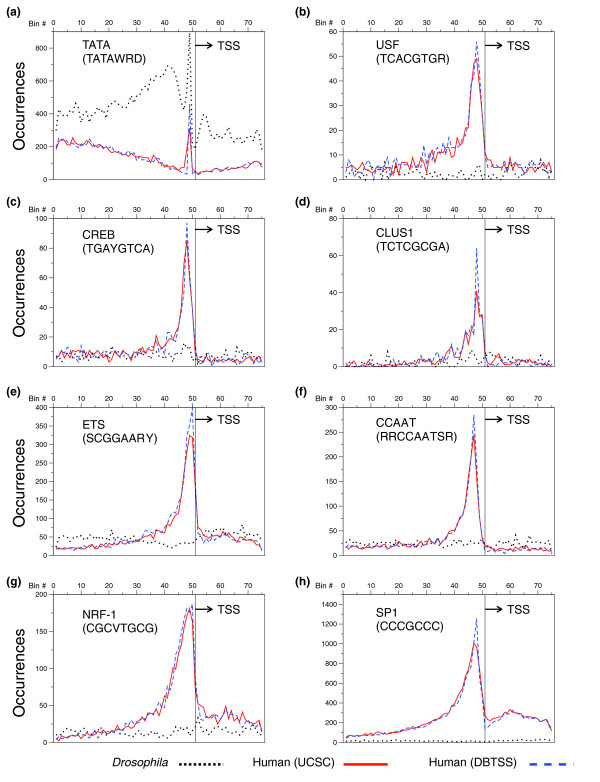
The distribution of 8 'human specific' motifs in *Drosophila *and human promoters. **(a-h) **The number of occurrences of each previously identified [11] human specific motif in each 20 bp bin for *Drosophila *(dotted black), human (UCSC; solid red) and human (DBTSS; dashed blue) promoters. The number of occurrences of each element has been normalized, based on a dataset of 10,000 promoters, to compensate for the different sizes of the datasets.

We examined if motifs that peak in *Drosophila *also peak in human and vice-versa. Of the 15 *Drosophila *motifs that peaked, four also localized in human promoters (TATA, INR, DPE1 and NDM2; Figure 8a,b,d,l) with INR, DPE1 and NDM2 occurring at much lower frequency in human promoters. While both the human and *Drosophila *promoters showed a clear overabundance of the CA dimer at the TSS (Figure 1d), we were previously [11] unable to detect an INR signal in human promoters using the degenerate human consensus sequence (YYANWYY). However, mapping the *Drosophila *INR motif (TCAGTY) to human promoters does produce a weak peak at the TSS in the UCSC dataset and a more pronounced peak in the DBTSS dataset (Figure 8b). Analysis of this peak at a 1 bp resolution (Figure 8x) revealed that both human datasets contain significantly fewer of these precisely positioned elements than does the *Drosophila *dataset. This result suggests that this TCAGTY motif plays a less significant role in human gene transcription than it does in *Drosophila*, and agrees with previous findings that the human INR is more degenerate than its *Drosophila *counterpart. It should be noted that in all cases, the motifs that contained a peak in one human dataset also showed peaks in the other human dataset, although the DBTSS dataset showed more pronounced peaks. This confirms both the qualitative similarity of the two datasets and the suggestion that the DBTSS data contains greater numbers of accurately positioned TSSs. Of the eight motifs previously identified to abundantly peak in humans [11], only TATA also peaked in *Drosophila *promoters (Figure 9).

In comparing the distributions of the *Drosophila *and human motifs, it is apparent that some sequences, even when they occur outside of the peak, display different abundances for the two organisms. This is true for DRE (Figure 8n), which peaks in *Drosophila *but is also a highly abundant motif outside of the peak (total of 7,058 across 1,500 bp of 10,914 promoters). In humans, there is no indication of any clustering, and this element is also very rare (total of 1,015 across 1,500 bp of 15,011 promoters). The reciprocal observation is made for human promoters, where SP1 (Figure 9h) is characterized by a very large peak and is also abundant outside of the peak but is virtually absent from *Drosophila *core promoters. In contrast, the INR (Figure 8b), which peaks in both organisms, albeit on different scales, shows very similar total abundance in both organisms (a total of 17,377 and 20,320 occurrences across 1,500 bp, in 10,914 and 15,011 promoters, for *Drosophila *and human, respectively).

### E-box motifs that peak in both *Drosophila *and humans

**Figure 10:**
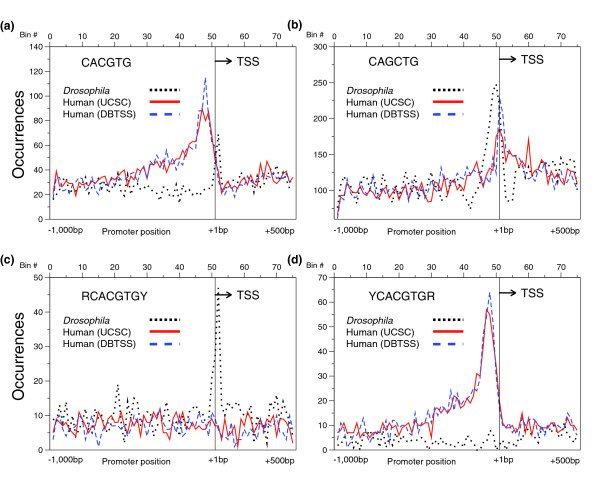
E-box variants that peak in *Drosophila *and human promoters. **(a-d) **The number of occurrences of **(a) **CACGTG,**(b) **CAGCTG, **(c) **RCACGTGY and **(d) **YCACGTGR in each 20 bp bin for *Drosophila *(dotted black), human (UCSC; solid red), and human (DBTSS; dashed blue) promoters.

NDM5 (CAGCTSWW) is a derivative of the general DNA sequence termed an E-box (CANNTG) that is bound by B-HLH-ZIP transcription factors, including the oncogene Myc|Max. A recent paper [18] has shown that an E-box sequence is located near the TSS of *Drosophila *genes. The sequence CACGTG is the core of the upstream stimulatory factor (USF) sequence previously identified in humans to peak near the TSS [11]. We compared the distribution of these related sequences in *Drosophila *and human. The USF consensus sequence (TCACGTGR) does not show any clustering in *Drosophila *(Figure 9b). However, the 6-mer E-box variants CACGTG and CAGCTG have peaks in both human and *Drosophila *promoters (Figure 10a,b). In *Drosophila*, the sequence CACGTG peaks downstream of the TSS while in human it peaks upstream of the TSS. The E-box variant CAGCTG peaks in both human and *Drosophila *just upstream of the TSS. Figures 9c,d highlight two E-box 8-mer variants with dramatically different peaking properties where sequences outside a conserved 6-mer define the peaking properties of the 8-mer. The sequence RCACGTCY peaks only in *Drosophila *while YCACGTGR peaks only in human, suggesting that distinct B-HLH proteins bind these related sequences.

### Correlation of different DNA motifs in the same promoter

We examined correlations in the occurrence of the 15 peaking motifs in *Drosophila *to gain insight into their potential combinatorial or redundant function. Table 1 presents a matrix showing: the number of promoters that contain one motif in a peak that also contain a second motif in a peak (a); the frequency of this co-occurrence (b); and the probability (c). There is a complex pattern of positive and negative correlation for individual motifs, suggesting that combinations of motifs act to regulate core promoter function.

For the precisely positioned directional motifs (DMp1 to 5: TATA, INR, INR1, DPE, and DPE1), promoters that contain INR also preferentially contain either the TATA or DPE sequence. However, TATA and DPE motifs negatively correlate. All five members of the DMp class negatively correlate with some or all of the DMv class. DMp1 to 5 positively correlate with three of the NDMs (NDM1 to 3) but negatively correlate with NDM4 and NDM5.

The five variably positioned directional motifs (DMv1 to 5) have both positive and negative correlations amongst themselves and with the NDMs. The DMv class members positively correlate with NDM4 and NDM5 and negatively correlate with NDM1 to 3, correlations that are exactly the opposite of those observed for the DMp class (see above). On average, members of the NDM class positively correlate with each other. Positive correlations between motifs suggest the possibility of physical interactions between the proteins that bind the co-occurring DNA motifs. Negative correlations, as are observed between the precisely positioned DMs (DMp) and the variably positioned DMs (DMv), suggest that the proteins that bind them have distinct functions.

### Consensus DNA motifs correlate with biological function

**Figure 11:**
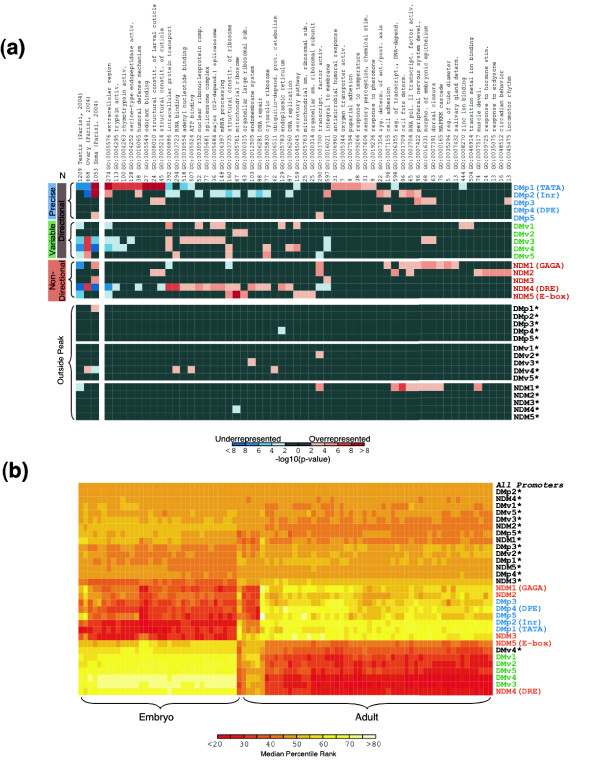
Correlations between DNA motifs in promoters and function (GO terms and mRNA expression properties). In both sections of the figure, promoter lists in blue are DMp, green are DMv, and red are NDM. Control groups with the DNA motifs not in the peak but between -1,000 bp and +499 bp are in black with an asterisk.**(a) **False-color image of representation bias in GO terms and mRNA expression clusters for the 15 DNA motifs, either in the peak or elsewhere in the promoter region. Values plotted are -log_10_(*p *value) calculated by Fisher's exact test. Data for the 54 most strongly correlated GO terms are shown (some redundant GO terms are removed). On the far left are results for over/under representation in self-organizing map (SOM) clusters identified from previously published expression data [20]. Over-represented categories are colored in red and under-represented categories are in blue. N values displayed at the top are total numbers of genes in the reference set assigned to that group. **(b) **False-color image of hierarchically clustered median percentile ranks of mRNA expression ratios, for previously published data for embryo and adult samples [21]. Each ratio represents expression relative to a global mean across arrays. Columns represent each of 89 array experiments, clustered so that embryo samples are at left and adult samples are at right. 'All Promoters' represents all genes and shows no preferences (median percentile rank = 50).

The non-random distribution of individual motifs and motif combinations at core promoters strongly suggests that the identified motifs are biologically significant and promoters that share the same motif in a peak may also share similar biological functions. To evaluate this possibility, we calculated statistical over- and under-representation of 5,200 Gene Ontology (GO) annotation terms [19] for *Drosophila *genes whose promoters contained any of the 15 motifs, either within the peak or elsewhere in the promoter region. We found highly significant correlations (*p *< 10^-4^) for each motif only when they occurred in the peak (Figure 11a). With one exception, the simple presence elsewhere within the 1,500 bp promoter region does not correlate with GO terms, demonstrating that the position of a motif in the promoter is critical for predicting biological function, as was observed in human promoters [11]. The directional positioned motifs, DMp and DMv, not only co-occur in promoters with either NDM1 to 3 or NDM4 and NDM5, respectively, but also correlate with similar GO terms. This indicates a combinatorial code of motifs at core promoters directing batteries of genes.

Additional insight can be inferred by examining individual GO terms that correlate. For example, *Drosophila *mitochondrial ribosomal genes contain the E-box (*p *< 10^-8^). In contrast, promoters of human mitochondrial ribosomal genes contain the ETS motif, a motif that peaks in human but not in *Drosophila*. Thus, even though the mitochondrial ribosomal genes are highly conserved, their regulation is evolving.

If core promoter motifs are used to drive the expression of gene batteries participating in a common biological process, this should be evident in global gene expression profiles. We turned to *Drosophila *mRNA expression patterns determined by micoarray experiments [20,21] to evaluate whether genes that are co-expressed have the same motif in their promoters. Figure 11a shows correlations between all 15 motifs, either in the peak or elsewhere in the promoter region, and gene expression in testis (male germline), ovary (female germline), and soma. The presence of TATA in the peak in the promoter positively correlates with gene expression in somatic tissue but negatively correlates with expression in germline tissue. The presence of positioned DMv3 to 5, and DRE in promoters positively correlates with female germline expression and negatively correlates with male germline expression. If the motif occurs outside the peak, few correlations are observed, supporting the conclusion that motif position is functionally important.

We see more striking correlations between promoter motifs and mRNA expression in the embryonic and adult stages of *Drosophila *development that express different sets of genes. Figure 11b presents a hierarchal clustering of mRNA expression for 89 samples from a survey of gene expression in embryos and adults for promoters containing any of the 15 motifs (either in or outside the peak). Genes with motifs in the peak show strong mRNA expression differences between embryo and adult samples, suggesting that these motifs help direct the differential utilization of the genome between embryos and adult. Genes with promoters containing DMv1 to 5 and co-occurring NDM4 and NDM5 are preferentially active in the embryo. In contrast, genes with promoters containing the three abundant precisely positioned directional motifs (TATA, INR, and DPE) and the co-occurring NDM1 to 3 are preferentially active in the adult.

### INR derivatives

**Figure 12:**
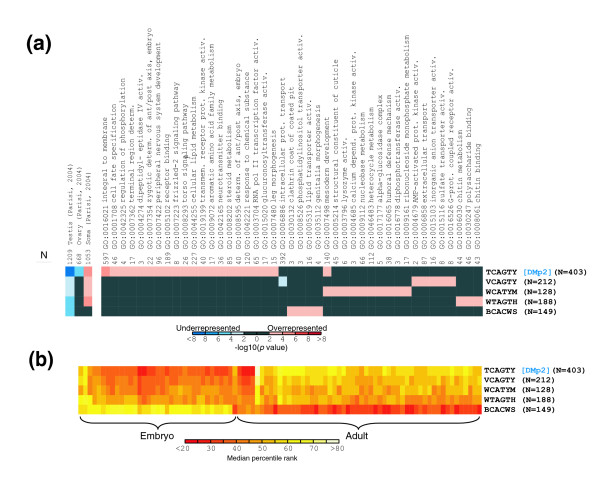
Correlations between five INR variants localized exactly at the TSS in promoters and function (GO terms and mRNA expression properties). **(a) **False-color image of representation bias in GO terms and mRNA expression clusters for the five variants of the INR motif in the peak. Values are calculated and displayed as in Figure 11a. The 42 most strongly correlated GO terms are shown. Note that each INR variant correlates with different GO terms. **(b) **False-color image of hierarchically clustered median percentile ranks of mRNA expression ratios, for previously published data for embryo and adult samples 21. Data are calculated and displayed as in Figure 1

Both *Drosophila *and human promoters have a CA peak exactly at the TSS in a significant number of promoters. About 2,100 *Drosophila *promoters contain the CA sequence at the TSS but only 400 of these are part of the consensus INR sequence (TCAGTY). We examined the remaining promoter sequences for related INR sequences and identified 4 more motifs, resulting in 1,080 promoters with INR related sequences exactly positioned at the TSS. To evaluate if these INR related sequences correlate with distinct functions or are variants of a single motif, we investigated the correlation of the INR variants with different biological properties by examining GO terms and mRNA expression properties. Figure 12a shows that the variant INR motifs have distinct patterns of enrichment with categories of GO terms. Similarly, the developmental mRNA expression analysis (Figure 12b) indicates that one of the INR motif variants (BCACWS) is preferentially associated with genes with embryonic expression while the other variants are preferentially associated with adult expression genes. While some of the GO categories enriched for specific INR variants (for example, mesoderm development) appear at odds with the adult/embryo expression patterns, the overall impression suggests that these variant INR sequences are functionally distinct and may be recognized by distinct proteins. The discrepancies between the GO term enrichment and adult/embryo expression patterns can be explained if one assumes that the preferential use of INR signals is not absolute. Thus, even though there is a general trend toward preferential use of different elements at different stages in development, certain genes may use the 'adult INRs' during embryogenesis.

## Discussion

We have determined the localization of all 8-mers in 10,914 *Drosophila *and two sets of human promoters (UCSC, 15,011 promoters; DBTSS, 12,926 promoters) aligned relative to the TSS and have identified DNA motifs that are non-randomly distributed in each dataset. Though we examined the region between -1,000 bp and +499 bp, all peaks are within 100 bp of the TSS. Two dramatic differences are observed between *Drosophila *and human promoters. First, there is little overlap in the DNA motifs that localize in the promoters of these two species. Second, of the 15 motifs identified in *Drosophila *promoters, 10 are directional DNA motifs (DNA sequences that occur on the positive but not the negative strand of DNA), while in human, promoters TATA, INR and DPE1 are the only DMs. We suggest that these DMs may be binding sites for core promoter selectivity factors [5]. While there is little overlap between motifs identified in *Drosophila *and human, both organisms contain identifiable TATA and INR core promoter elements, with humans having only a barely discernable DPE element. The identification of common elements in both species indicates a fundamental similarity in core promoter organization, as would be expected because the proteins that bind these sequences are conserved in both species.

A comparison of the promoter structures of two organisms depends on the quality of the data being analyzed. In an attempt to ensure that our results were not biased by differences in the quality of annotation of the TSS of the *Drosophila *and human genomes, we have analyzed three datasets. We used the annotation from the UCSC Genome Browser for both *Drosophila *and human to construct a dataset of promoters that represents the standard view of these genomes. Additionally, we have constructed a set of promoters based on annotations from the human DBTSS [22], a database specifically aimed at correctly identifying the TSS through the use of full-length cDNA cloning methods. As shown in Figure 1d, all three datasets show distinct CA peaks at the TSS, with the *Drosophila *peak being intermediate in amplitude between the two human datasets. The qualitative similarity of the findings of the two human datasets suggests that the differences we observe between the *Drosophila *and human promoters are not due to differences in the quality of the underlying datasets. Additionally, the fact that both *Drosophila *and human datasets are sufficiently aligned with respect to the TSS is exemplified by our ability to readily identify over-represented, localized 8-mers in all datasets. We note that our technique is aimed at finding abundant over-represented, localized motifs that have a low degree of degeneracy. Thus, our inability to find a given motif in an organism could indicate one of four possibilities: the motif is absent; the motif is present in low abundance; the motif is present but is highly degenerate; or the motif is present but not significantly constrained with respect to its position relative to the TSS.

Previous work has addressed the DNA sequence of *Drosophila *promoters. However, these studies have either examined a limited number of promoters or did not examine the position of motifs relative to the TSS. Kutach and Kadonaga [23] examined a set of 200 *Drosophila *promoters and identified four types of promoters characterized by containing TATA only (29%), DPE only (26%), TATA + DPE (14%), or neither DNA motif (31%). Our global analysis looks at a much larger set of *Drosophila *promoters and finds a lower proportion of genes with these sequences. Instead of 60% of promoters containing a TATA motif, we find only 4.7% and, instead of 40% of promoters containing a DPE motif, we find only 2.1% of promoters that contain these motifs. Kutach and Kadonaga [23] used a less stringent criterion to define the motifs and it is also possible that the 200 promoters examined were biased towards TATA and DPE. They observed a conserved distance between the INR and DPE motifs and experimentally demonstrated that the conserved distance is critical for optimal function. This conserved distance is confirmed in our global analysis.

Another analysis of 2,000 *Drosophila *promoters identified 10 motifs that are conserved near the TSS [10]; we identified 15 motifs, including 9 of the 10 identified by Ohler *et al*. The motif that did not peak in our analysis is motif ten element (MTE), a downstream element important for initiation [24]. Our global analysis extends this analysis of 2,000 promoters. We show that many of the identified DNA motifs occur on only one strand of DNA and are uniquely positioned relative to the TSS. Furthermore, the DNA sequences that peak in *Drosophila *are different from the DNA sequences that peak in human promoters.

### Variably positioned directional motifs may be bound by core promoter selectivity factors

There has been little systematic analysis of *Drosophila *promoter function as it relates to regulation versus basal activity. One potential mechanism of regulated gene expression is for the RNA polymerase II complex to use different components in different promoters. This system is used in prokaryotic cells where sigma factors bind different DNA sequences that are part of the polymerase binding site and consequently regulate different sets of genes. Such factors in eukaryotic systems are termed core promoter selectivity factors [5]. Several properties might be expected for DNA motifs bound by core promoter selectivity factors: they occur on one strand of DNA, thus providing directional information to polymerase; they are precisely positioned relative to the TSS; binding sites for different core promoter selectively factors negatively correlate with each other in the same promoter; and the motifs should positively correlate with genes with a similar function. The precisely positioned DMp1 to 5 display all four characteristics while the variably positioned DMv1 to 5 match all criteria except that they are not uniquely positioned. Biochemical studies have already identified the DMp1 to 5 motifs as core promoter motifs (TATA, INR, DPE). We suggest that DMv1 to 5 may also be core promoter motifs that function independently of the DMp1 to 5 motifs. The DMv motifs are preferentially used in the embryo while the DMp motifs are used in the adult, consistent with an earlier suggestion that the mechanism of gene expression is different in the embryo than in the adult [21]. The DMv class of motif is not observed in humans and has not been studied biochemically.

When examining all aligned promoters, the most distinct feature is the TSS, which is observed even when we examine the distribution of the four mono-nucleotides at a single base-pair resolution. The CA dinucleotide sequence has a peak exactly at the TSS containing approximately 2,100 members of which approximately 1,400 members are above background. Of these, only 29% have the INR consensus TCAGTY. We defined four additional variant INR motifs that represent another 35% of the CA dinucleotides, indicating that two-thirds of the CA dinucleotides at the TSS are INR or variant motifs. In theory, the INR variants might all have the same general function. However, these variant INR motifs have distinct and nearly non-overlapping enrichments with specific GO terms. Furthermore, genes with one INR variant (BCACWS) are preferentially expressed in the embryo, instead of the adult. These associations with GO terms and different expression patterns demonstrate that variant INR motifs are biologically distinct and suggest they may be bound by different proteins or modified proteins in addition to the proteins known to bind the consensus INR (for example, RNA polymerase, TFIID, TBP250, and TFII-I [1]). It will be interesting to experimentally determine whether known INR binding proteins have different affinities for the five INR variants.

### Gene regulation in *Drosophila *and humans

Two observations suggest that *Drosophila *and human promoters use different mechanisms to regulate gene expression. First, they have a different frequency and distribution of mononucleotides in promoters. This distribution correlates with nucleosome positioning. Second, *Drosophila *promoters have a large number of DMs near the TSS while they are nearly absent from human promoters.

*Drosophila *promoters are A and T rich with a peak of A and T dinucleotides between -200 bp and the TSS (Figure 1), a region that experimentally is known to be nucleosome free, particularly for active genes [25]. A similar correlation is observed in the yeast genome where the promoter regions between -200 and the TSS are A and T rich and devoid of nucleosomes [26]. In *Drosophila*, the transcription factors that bind NDM1 to 5 bind in this nucleosome free region and could interact with the pre-initiation complex composed of RNA polymerase and proteins that bind the DMs (DMp or DMv) that are critical for defining the TSS. This model of promoter organization has an appealing simplicity. The promoter region is accessible and is regulated by complex interactions between proteins that bind different DNA sequences; NDMs in the core promoter, DMs that act as core promoter selectivity elements, and distant enhancers.

In humans, the core promoter is different so the above model does not apply. There is no nucleosome free region observed in promoters [27] and this is consistent with a valley in A and T distribution at the TSS. Upstream of the TSS are NDMs, binding sites for transcription factors that recruit cofactors involved in chromatin remodeling. A simple image is that chromatin remodeling displaces the nucleosome over the TSS, leaving naked DNA that is the signal for polymerase initiation. This model would explain the absence of DMs in human promoters. The core promoter elements are more degenerate in human, suggesting that the energy for binding of the general transcriptional machinery comes from more global architectural features of the promoter.

Perhaps the differences in *Drosophila *and human promoter architecture reflect a solution to the over 10-fold larger size of the human genome (2.9 × 10^9 ^bp) compared to the *Drosophila *genome (1.8 × 10^8 ^bp). It has been suggested that repression of inappropriate gene expression is more important as a genome becomes larger [28]. Thus, it may be that the critical step in human gene regulation is relieving repression by displacing the nucleosome over the TSS while in *Drosophila *it is the assembly of the components that bind specifically to the DNA motifs in the promoter. This may also help explain the evolution in vertebrates of a G and C rich region over the TSS that contains CpG islands that can be repressed by methylation [29]. Such methylation is greatly reduced in *Drosophila*.

### Core promoter structure evolves rapidly

The only DNA motifs that peak in *Drosophila *and human promoters are TATA, INR, DPE, NDM2, and the E-box. Conservation of motifs might be expected to occur in highly conserved genes, thus we examined whether the evolutionarily conserved mitochondrial ribosomal genes that function in a large multi-protein complex had similar DNA motifs in *Drosophila *and human promoters. The ETS motif is found in the promoters of human [11] and other mammalian mitochondrial ribosomal genes [30]. In *Drosophila*, the ETS motif does not occur in these promoters, even though the ETS protein is present in the *Drosophila *genome. In contrast, the E-box sequence clusters in *Drosophila *mitochondrial ribosomal genes. This highlights the observation that even for genes that are conserved over a long evolutionary time, the DNA motifs that regulate them are not always conserved. Similarly, there is a fast turnover of DNA sequences controlling the expression of ribosomal protein genes in different species of yeast [31] and the recent genome wide comparison of human and chimpanzee showed that regulatory sequences were the most rapidly evolving part of the genome [32].

The failure to find similar positioned motifs in human and *Drosophila *would be trivial if the DNA binding proteins were absent in one of the species. This does not appear to be the case. In many cases where DNA motifs peak in human promoters but not in *Drosophila *promoters, the proteins that bind them are present in *Drosophila*. For example, the CRE motif peaks in human but not in *Drosophila *promoters. However, CREB and other B-ZIP proteins that bind the CRE sequence (5'-TGACGTCA-3') are conserved between the two species [33] and genetic mutation of these loci produce dramatic phenotypes, demonstrating their functional importance. This suggests either that the signaling and transcriptional pathways are operating but are not regulating enough genes to produce a peak in the distribution, or the transcription factors can function at a variable distance from the TSS, or the motifs are so highly degenerate that they do not produce an identifiable signature. As more genomes are sequenced and DNA motifs identified that peak in promoters, it will become more obvious how transcription factors are used in evolution to express coordinately regulated genes. Our data support the emerging notion that evolution of gene regulation underpins many of the differences between species. These changes in gene expression are mediated in part by sequences located very close to the TSS.

## Conclusion

We used the technique of determining the non-random distribution of DNA sequences to identify 298 8-mers with highly significant (*p *≤ 1 × 10^-16^) distribution patterns in a set of 10,914 *D. melanogaster *promoters. These sequences were grouped into 15 unique motifs that were further classified into three families: precisely positioned DMs (DMp1 to 5); variably positioned DMs (DMv1 to 5); and NDM1 to 5. Correlations between GO annotation and mRNA expression patterns suggest that these different motifs play different functional roles. Additionally, we suggest that the DMs may be binding sites for core promoter selectivity factors in *Drosophila*. A comparison of the promoter regions of *Drosophila *and human revealed two characteristics that suggest that they use different mechanisms to regulate gene expression. First, the frequency and distribution of mononucleotides in *Drosophila *and human promoters are markedly different. Second, we have identified a large number of DMs near the TSS of *Drosophila *while the only identifiable DMs in human promoters are TATA, INR, and DPE. Thus, these data support the emerging notion that evolution of gene regulation underpins many of the differences between species.

## Materials and methods

### Dataset generation

Genomic DNA sequence and gene annotation data for *Drosophila *(Jan 2003, dm1), human (May 2004, hg17) were downloaded from the UCSC Genome Browser site [13,34]. For each organism a dataset was generated that contained only those RefSeq genes that had a unique transcription start site and at least 10 bp separating the TSS and the translation start site (ATG). When multiple RefSeq entries were identified as being identical by *blastclust *[35], a single entry was used to represent that region. While frequently ignored (masked) in promoter analyses, we have not excluded repetitive sequences in this study. For each entry the 1,500 bp corresponding to the region -1,001 to +499, relative to the TSS, was extracted from the genomic sequence data and subjected to the analyses describe in this manuscript. The total number of promoters represented in each dataset was 10,914 for *Drosophila *and 15,011 for human (UCSC). In addition, a second human dataset was prepared using the DBTSS annotations [14], and hg17 sequence data. A 1,500 bp promoter dataset was generated for the 5'-most TSS of each DBTSS annotated gene cluster. Entries that had an annotated ATG, translation start site, within 30 bp of the TSS were rejected. The resulting human (DBTSS) dataset contains 12,926 promoters.

### Analysis

The datasets were queried with the programs *fuzznuc *from the EMBOSS suite of software [36] or *tacg *[37] to locate the occurrence and position of different DNA sequence motifs.

### 8-mer/6-mer analysis

The raw data generated by *tacg *was processed by a combination of scripts and programs to generate the final binned distribution for each 8-mer/6-mer. To analyze the data, we divided the 1,500 bp into 75 bins, with each bin containing 20 bp. For the dataset -1,000 bp to +499 bp the numbering for bin 1 is -1,000 bp to -981; thus, bin 51 is from +1 bp to +20 bp. We determined the number of times a particular DNA sequence occurred in each 20 bp bin. The *Drosophila *distribution pattern for each 8-mer along with the identity of the promoters containing each 8-mer is available [38].

### Clustering factor calculation

To determine if a DNA sequence forms a peak in its distribution (that is, clustered), we used an automated method of detecting and quantifying peak height. For the 75 bins in each frequency distribution a mean () and standard deviation (*σ*) were determined. Those points, which were ≥2 standard deviations above the mean, were considered to be part of the peak and a new mean () and standard deviation (*σ*') were calculated excluding these points. The CF was then calculated based on the maximum bin value (*x*_*max*_) and the corrected mean and standard deviation:



### Calculation of *P *value for distribution

To evaluate the probability that the clustering results were obtained by chance, we converted the CF values into probability terms based on the analysis of the occurrence of each 8-mer in 1,000 random datasets as described previously [11]. We generated 1,000 random datasets, each containing 10,914 sequences 1,500 bp long, using the 8-mer frequencies observed in the original Drosophila dataset. Finally, we calculated the probability term, *P*, that represents -log_10_(1 - *p*), where *p *is the area that lies under the normalized curve of the distribution of CF_expt_. Thus, the greater the P value the more unlikely it is that the result could occur by chance.

The clustering and graphing of the data were performed using the programs Microsoft Excel and/or Grace [39].

### Sequence logos

Graphical representations of the 15 *Drosophila *motifs, in the form of sequence logos [40], were generated using the WebLogo software [41].

### Calculation of *P *value for subsets in a set

To determine the significance of the numbers presented in Table 1, we calculated two-tailed normalized cumulative probability (*P *value) that the numbers were greater (or less) than expected by random chance. The number of possible associations of *s*_*2 *_elements out of *S *elements is:



the number of combinations when subsets *s*_*1 *_and *s*_*2*_have *m *members in common:



and the probability of having *m *members in the intersection



where  is combinatorial combination.

The cumulative probability that the value m* is greater or less than expected is, respectively,



or



where *m*_*max *_= min (*s*_1_,*s*_2_). We doubled the result so cumulative probability varies in a range *0 *to *1*, and took the logarithm:

*P*(m*) = -*Log*_10_(*I*)

The value of *P *indicates the statistical probability of numbers occurring by chance: the greater the number, the more statistically significant the result.

### GO term analysis

Patterns in gene product functions for each promoter group were investigated using their assignments to GO terms. Each of the 4,192 GO terms in the *Drosophila *GO annotation, and their 1,008 parent GO terms (5,200 total), was analyzed. Gene group NM identifiers were matched up to Flybase identifiers by retrieving CG numbers from a batch GenBank search, and then matching up with FBgn identifiers through Flybase. GO assignments were retrieved from a flat file downloaded from the 'current annotations' page at the Gene Ontology website [42]. File update is dated 18 June 2005. Since our promoter analysis was based on an earlier annotation, the complete set of GO annotations was reduced to create a normalized reference. GO annotations for FlyBase identifiers that are not included in the original set of promoters were removed. Dependencies for each GO term were retrieved using the R package GOstats. The number of occurrences for each gene list matched up to a GO term of interest or its children were counted, and considered the observed value. The expected value was calculated as: (number of genes assigned to GO term and children/the number of genes in the entire normalized reference) × the number of genes in the group with the GO annotation. This expected value was used to calculate the O/E ratio. These values were used to convert *P *values to a positive or negative value to indicate correlation direction. *P *values were generated with a 2 × 2 matrix for each promoter group/GO term pair [43] with the fisher.test function in R [44].

### mRNA expression correlation with motifs that peak

Promoter lists were correlated with mRNA expression patterns that vary by sex, developmental stage, and tissue by examining microarray results from previous publications [20,21]. Testis, ovary, and soma-biased expression were categorized by performing hierarchical clustering, generating gene lists that occur in the same self-organizing map (SOM) cluster [20]. Observed and expected representation of each promoter class and *P *values were calculated by 2 × 2 fisher exact test in a similar fashion to the GO term analysis described above. Adult and embryo expression patterns [21] were examined by calculating the median rank of all expression values in each sample, and performing hierarchical clustering. In each case, a standardized reference was created that corrected for differences in annotation and microarray platform.

## Figures and Tables

**Table 1:** The co-occurrence in the same promoter of DNA motifs that cluster

	**Motif**			**DMp1**	**DMp2**	**DMp3**	**DMp4**	**DMp5**	**DMv1**	**DMv2**	**DMv3**	**DMv4**	**DMv5**	**NDM1**	**NDM2**	**NDM3**	**NDM4**	**NDM5**
	**Ohler no.**			**3**	**4**		**9**			**8**	**7**	**1**	**6**				**2**	**5**
	**Name**			**TATA**	**INR**	**INR1**	**DPE1**	**DPE2**						**GAGA**			**DRE**	**E-box**
	**Totals**		**8289**	**511**	**1501**	**113**	**80**	**147**	**311**	**311**	**604**	**649**	**287**	**359**	**424**	**215**	**1593**	**1184**
**(a)**	STATAAA	DMp1	511		98	9	2	4	2	8	10	**6**	4	19	28	9	**21**	**26**
	TCAGTY	DMp2	1501	98		12	25	**43**	**15**	**18**	**34**	**17**	**12**	**100**	**108**	38	**67**	**112**
	TCATTCG	DMp3	113	9	12		0	5	3	2	2	4	1	10	5	5	9	9
	CGGACGT	DMp4	80	2	25	0		1	1	2	4	2	2	10	6	1	6	9
	KCGGTTSK	DMp5	147	4	**43**	5	1		3	0	2	4	3	14	11	7	**4**	18
	CARCCCT	DMv1	311	2	**15**	3	1	3		16	13	18	6	5	7	7	**79**	46
	TGGYAACR	DMv2	311	8	**18**	2	2	0	16		8	15	6	4	6	6	59	**64**
	CAYCNCTA	DMv3	604	10	**34**	2	4	2	13	8		18	9	**1**	16	9	**282**	63
	GGYCACAC	DMv4	649	**6**	**17**	4	2	4	18	15	18		**64**	8	12	12	95	59
	TGGTATTT	DMv5	287	4	**12**	1	2	3	6	6	9	**64**		0	5	2	26	38
	GAGAGCG	NDM1	359	19	**100**	10	10	14	5	4	1	8	0		26	18	**6**	28
	CGMYGYCR	NDM2	424	28	**108**	5	6	11	7	6	16	12	5	26		6	33	34
	GAAAGCT	NDM3	215	9	38	5	1	7	7	6	9	12	2	18	6		22	33
	ATCGATA	NDM4	1593	**21**	**67**	9	6	**4**	**79**	59	**282**	95	26	**6**	33	22		**265**
	CAGCTSWW	NDM5	1184	**26**	**112**	9	9	18	46	**64**	63	59	38	28	34	33	**265**	
	**Unique**		**4156**	**304**	**932**	**58**	**30**	**48**	**146**	**146**	**220**	**366**	**141**	**165**	**195**	**88**	**783**	**534**

	**Totals**		**8289**	**511**	**1501**	**113**	**80**	**147**	**311**	**311**	**604**	**649**	**287**	**359**	**424**	**215**	**1593**	**1184**
**(b)**	STATAAA	DMp1	511	4.7	6.5	8.0	2.5	2.7	0.6	2.6	1.7	**0.9**	1.4	5.3	6.6	4.2	**1.3**	**2.2**
	TCAGTY	DMp2	1501	19.2	13.8	10.6	31.3	**29.3**	**4.8**	**5.8**	**5.6**	**2.6**	**4.2**	**27.9**	**25.5**	17.7	**4.2**	**9.5**
	TCATTCG	DMp3	113	1.8	0.8	1.0	0.0	3.4	1.0	0.6	0.3	0.6	0.4	2.8	1.2	2.3	0.6	0.8
	CGGACGT	DMp4	80	0.4	1.7	0.0	0.7	0.7	0.3	0.6	0.7	0.3	0.7	2.8	1.4	0.5	0.4	0.8
	KCGGTTSK	DMp5	147	0.8	**2.9**	4.4	1.3	1.4	1.0	0.0	0.3	0.6	1.1	3.9	2.6	3.3	**0.3**	1.5
	CARCCCT	DMv1	311	0.4	**1.0**	2.7	1.3	2.0	2.9	5.1	2.2	2.8	2.1	1.4	1.7	3.3	**5.0**	3.9
	TGGYAACR	DMv2	311	1.6	**1.2**	1.8	2.5	0.0	5.1	2.9	1.3	2.3	2.1	1.1	1.4	2.8	3.7	**5.4**
	CAYCNCTA	DMv3	604	2.0	**2.3**	1.8	5.0	1.4	4.2	2.6	5.5	2.8	3.1	**0.3**	3.8	4.2	**17.7**	5.3
	GGYCACAC	DMv4	649	**1.2**	**1.1**	3.5	2.5	2.7	5.8	4.8	3.0	6.0	**22.3**	2.2	2.8	5.6	6.0	5.0
	TGGTATTT	DMv5	287	0.8	**0.8**	0.9	2.5	2.0	1.9	1.9	1.5	**9.9**	2.6	0.0	1.2	0.9	1.6	3.2
	GAGAGCG	NDM1	359	3.7	**6.7**	8.9	12.5	9.5	1.6	1.3	**0.2**	1.2	0.0	3.3	6.1	8.4	**0.4**	2.4
	CGMYGYCR	NDM2	424	5.5	**7.2**	4.4	7.5	7.5	2.3	1.9	2.7	1.9	1.7	7.2	3.9	2.8	2.1	2.9
	GAAAGCT	NDM3	215	1.8	2.5	4.4	1.3	4.8	2.3	1.9	1.5	1.9	0.7	5.0	1.4	2.0	1.4	2.8
	ATCGATA	NDM4	1593	**4.1**	**4.5**	8.0	7.5	**2.7**	**25.4**	19.0	**46.7**	14.6	9.1	**1.7**	7.8	10.2	14.6	**22.4**
	CAGCTSWW	NDM5	1184	**5.1**	**7.5**	8.0	11.3	12.2	14.8	**20.6**	10.4	9.1	13.2	7.8	8.0	15.4	**16.6**	10.9
	**Unique**			**59.5**	**62.1**	**51.3**	**37.5**	**32.7**	**47.0**	**47.0**	**36.4**	**56.4**	**49.1**	**46.0**	**46.0**	**40.9**	**49.2**	**45.1**

	**Totals**		**8289**	**511**	**1501**	**113**	**80**	**147**	**311**	**311**	**604**	**649**	**287**	**359**	**424**	**215**	**1593**	**1184**
**(c)**	STATAAA	DMp1	511		3.2	0.8	0.3	0.5	4.1	1.1	4.1	**7.3**	2.4	0.2	1.1	0.1	**14.2**	**5.4**
	TCAGTY	DMp2	1501	3.2		0.4	4.1	**5.9**	**6.5**	**5.1**	**10.2**	**22.6**	**7.0**	**11.8**	**10.1**	0.9	**40.4**	**5.6**
	TCATTCG	DMp3	113	0.8	0.4		0.1	1.4	0.0	0.1	1.0	0.4	0.4	2.1	0.0	0.8	1.3	0.4
	CGGACGT	DMp4	80	0.3	4.1	0.1		0.0	0.2	0.0	0.0	0.6	0.0	3.3	0.7	0.0	1.1	0.0
	KCGGTTSK	DMp5	147	0.5	**5.9**	1.4	0.0		0.1	1.6	1.7	0.9	0.0	3.2	1.3	1.3	**5.5**	0.2
	CARCCCT	DMv1	311	4.1	**6.5**	0.0	0.2	0.1		1.5	0.5	0.0	0.2	1.0	0.8	0.1	**6.3**	1.5
	TGGYAACR	DMv2	311	1.1	**5.1**	0.1	0.0	1.6	1.5		1.8	0.3	0.2	1.4	1.1	0.0	1.4	**6.3**
	CAYCNCTA	DMv3	604	4.1	**10.2**	1.0	0.0	1.7	0.5	1.8		3.1	1.1	**7.4**	0.9	0.3	**84.2**	0.1
	GGYCACAC	DMv4	649	**7.3**	**22.6**	0.4	0.6	0.9	0.0	0.3	3.1		**19.9**	2.9	2.4	0.0	0.0	0.8
	TGGTATTT	DMv5	287	2.4	**7.0**	0.4	0.0	0.0	0.2	0.2	1.1	**19.9**		3.9	1.2	0.8	2.2	0.6
	GAGAGCG	NDM1	359	0.2	**11.8**	2.1	3.3	3.2	1.0	1.4	**7.4**	2.9	3.9		2.5	3.3	**16.8**	1.2
	CGMYGYCR	NDM2	424	1.1	**10.1**	0.0	0.7	1.3	0.8	1.1	0.9	2.4	1.2	2.5		0.3	4.7	1.2
	GAAAGCT	NDM3	215	0.1	0.9	0.8	0.0	1.3	0.1	0.0	0.3	0.0	0.8	3.3	0.3		1.1	1.3
	ATCGATA	NDM4	1593	**14.2**	**40.4**	1.3	1.1	**5.5**	**6.3**	1.4	**84.2**	0.0	2.2	**16.8**	4.7	1.1		**13.5**
	CAGCTSWW	NDM5	1184	**5.4**	**5.6**	0.4	0.0	0.2	1.5	**6.3**	0.1	0.8	0.6	1.2	1.2	1.3	**13.5**	

The 15 motifs are grouped into three groups, DMp1 to 5, DMv1 to 5, and NDM1 to 5. **(a) **The number of promoters that contain two motifs, each that occurs in a peak, was determined. To the left are the 15 motifs followed by the number of their occurrences in the peak. **(b) **The frequency of promoters containing one motif also containing a second motif. DMp1 (TATA) for example, is found in 4.7% of all promoters but occurs in 6.5% of promoters that contain DMp2 (INR). **(c) **The probability. Throughout all three panels of the table, positive correlations are shown as normal numbers, negative correlations are underlined and if the probability term has a value *p *≤ 10^-5^, one in 100,000, then the numbers are in bold. For example, INR is found in 1,501 promoters, which is 13.8% of all promoters. However, in the 1,593 DRE promoters, the INR only occurs in 4.2% of them. This observed under-representation or negative correlation has a one in 10^40 ^probability occurring by chance.
